# Asparagine-related biomarkers and regulatory mechanisms in type 2 diabetes mellitus

**DOI:** 10.3389/fmolb.2025.1733878

**Published:** 2025-12-17

**Authors:** Jiayi Xia, Tao Cai, Peiyin Chen, Lu Gan, Bo Cao, Mingming Kong

**Affiliations:** 1 Department of Endocrinology, The First Affiliated Hospital of Guizhou University of Traditional Chinese Medicine, Guiyang, Guizhou, China; 2 Department of Colorectal and Anal Surgery, The First Affiliated Hospital of Guizhou University of Traditional Chinese Medicine, Guiyang, Guizhou, China; 3 Department of Endocrinology, The Second People’s Hospital of Guizhou Provincial, Guiyang, Guizhou, China; 4 Outpatient Department, The 970th Hospital of PLA Joint Logistic Support Force, Yantai, Shandong, China

**Keywords:** type 2 diabetes mellitus, asparagine, protein phosphatase 1 catalytic subunit alpha, cathepsin D, immune infiltration

## Abstract

**Background:**

Type 2 diabetes mellitus (T2DM) is a complex metabolic disorder. Emerging evidence suggests asparagine metabolism might play a pivotal role in T2DM, yet the underlying molecular mechanisms remain elusive. This study aimed to detect asparagine-related biomarkers and expound their functional roles in T2DM pathogenesis.

**Methods:**

Transcriptomic datasets from peripheral blood samples of T2DM patients and controls were analyzed. Differential expression analysis, protein-protein interaction (PPI) network, and machine learning algorithms, followed by expression analysis across cohorts were employed to screen biomarkers. Biomarker diagnostic performance was evaluated. Functional enrichment, immune infiltration analysis, and multi-layer regulatory network construction were conducted. Drug-target interactions and molecular docking were explored to identify potential therapeutics.

**Results:**

A total of 90 candidate genes were detected. Four feature genes were screened via multi-algorithm integration. Protein phosphatase 1 catalytic subunit alpha (PPP1CA) and cathepsin D (CTSD) were validated as biomarkers, showing significant upregulation in T2DM samples and high diagnostic accuracy (AUC of PPP1CA = 0.969 and CTSD = 0.984 in the training cohort, AUC of PPP1CA = 0.806 and CTSD = 0.875 in the validation cohort, respectively). Functional enrichment highlighted distinct yet complementary functional roles of PPP1CA and CTSD in T2DM progression. Immune infiltration revealed elevated activated dendritic cells, mast cells, and myeloid-derived suppressor cells in T2DM samples, with PPP1CA and CTSD correlating significantly with these cell types. Regulatory networks identified shared transcription factors and miRNAs targeting both genes. Pharmacological screening prioritized norcantharidin and naringenin as high-affinity compounds targeting these biomarkers.

**Conclusion:**

This study identified PPP1CA and CTSD as asparagine-related biomarkers driving immune-metabolic crosstalk in T2DM. The príicted regulatory networks and therapeutic compounds provided novel insights into T2DM mechanisms and potential intervention strategies.

## Introduction

Type 2 diabetes mellitus (T2DM) represents one of the most problematic health issues worldwide in the 21st century. This complex, multisystemic metabolic disorder is characterized by chronic hyperglycemia resulting from a progressive defect in insulin secretion or tissue resistance to insulin (Młynarska et al., 2025; Adiga et al., 2021). Globally, an estimated 462 million individuals are affected by T2DM, corresponding to 6.28% of the world’s population (Khan et al., 2020). Between 1990 and 2022, the age-standardized prevalence of T2DM rose in 131 countries for women and in 155 countries for men, with a posterior probability exceeding 0.80. The most significant increases occurred in low- and middle-income nations across Southeast Asia, South Asia, the Middle East, North Africa, and Latin America Caribbean (NCD Risk Factor Collaboration, 2024). The pathogenesis of T2DM is primarily associated with insulin resistance (IR) and pancreatic β-cell dysfunction (Młynarska et al., 2025). Under insulin-resistant conditions, the sensitivity of peripheral tissues—such as skeletal muscle and adipose tissue—to insulin is diminished, leading to impaired glucose metabolism. Pancreatic β-cell dysfunction, in turn, results in inadequate insulin secretion that fails to meet the body’s metabolic demands, thereby inducing hyperglycemia (Lu et al., 2024). T2DM not only directly harms multiple organ systems but also worsens the risk and severity of cardiovascular diseases, renal failure, and related neuropathies, which severely diminish patients’ quality of life and shorten life expectancy (Pan et al., 2025). Therefore, in-depth investigation into the underlying pathogenic and molecular mechanisms of T2DM, along with the identification of molecular biomarkers and targeted therapeutic agents, is of paramount importance for improving the quality of life and prognosis of T2DM patients.

Asparagine is a crucial non-essential amino acids (AAs) that plays a pivotal role in protein synthesis and nitrogen metabolism. In recent years, the involvement of asparagine in metabolism-related diseases has garnered increasing scientific attention. Emerging evidence suggests a potential association between asparagine and T2DM (Chung et al., 2015; Newgard et al., 2009; Wang S. et al., 2022). In Sprague Dawley rats with T2DM, the metabolic pathway of aminoacyl-t-RNA biosynthesis was significantly upregulated. Meanwhile, AAs, including glycine, L-asparagine, and L-serine were also significantly increased in T2DM rats, which was prospectively associated with impaired insulin secretion and an increase in glucose levels (Ndlovu et al., 2023; Wang L. et al., 2022). In addition, the increased serum glutamic acid, lysine, phenylalanine, arginine, alanine, tyrosine, aspartic acid, in patients with T2DM were positively correlated with the metabolic disorder, such as fasting glucose, homeostasis model assessment of insulin resistance (HOMA-IR) (Lee et al., 2018). More notably, the ratio of asparagine to other AAs demonstrates greater predictive value for T2DM disease status and prognosis than individual AAs levels. When the ratio of asparagine and aspartate exceeds 1.5, the risk of T2DM significantly increases (OR = 7.99, 95% CI: 5.50–11.6), especially in females gender and by >50 years of age (Luo et al., 2020). All available evidence indicated that abnormal asparagine, a robust biomarker in T2DM, contributed to IR and defects of insulin secretion, and deteriorated T2DM pathogenesis. However, the specific mechanistic role of asparagine in T2DM pathogenesis remains incompletely elucidated. Further investigation into the involvement of asparagine in T2DM development is essential for uncovering novel pathogenic mechanisms and identifying potential therapeutic targets.

This present study systematically identifies key biomarkers associated with asparagine metabolism and elucidates their functional roles, transcriptional and epigenetic regulatory networks in the pathogenesis of T2DM. We integrated multiple approaches, including transcriptomic data analysis, machine learning, and experimental validation. Additionally, potential therapeutic agents targeting these biomarkers were explored. Analysis of the workflow is presented in Supplementary Figure S1. Our study provides novel molecular mechanisms insights into asparagine’s involvement in T2DM, enriching the theoretical framework of diabetic etiology. Findings from this study hold promise for optimizing clinical management strategies and advancing novel therapeutics against T2DM.

## Materials and methods

### Ethics approval and consent to participate

This study was approved by the ethics committee of the First Affiliated Hospital of Guizhou University of Traditional Chinese Medicine (Number: KL2024-017). All participants provided written informed consent prior to the study. A total of 10 newly diagnosed patients with T2DM, aged 40–65 years (mean age: 47.23 ± 7.41 years), and 10 healthy individuals, aged 40–65 years (mean age: 47.23 ± 7.41 years), were included in this study. The diagnosis of T2DM was established based on the criteria set by the American Diabetes Association (ElSayed et al., 2023). Participants were recruited from individuals attending our hospital. Participants with acute or chronic inflammatory diseases, cardiovascular disease, uncontrolled hypertension, type 1 diabetes mellitus (T1DM), gestational diabetes, smoking habits, or alcohol consumption were excluded.

### Real-time quantitative PCR for mRNA expression in peripheral blood mononuclear cells

Peripheral blood mononuclear cells (PBMCs) were isolated from heparinized whole blood samples immediately using Ficoll-Hypaque density-gradient centrifugation under sterile conditions as previously conducted (Arab Sadeghabadi et al., 2022). After washing the cells twice with phosphate-buffered saline, isolated PBMCs were suspended in RPMI 1640 medium. Then PBMCs were counted, 5 × 10^6^ cells/well plated in a 6-well plate containing RPMI 1640 supplemented with 10% fetal bovine serum and 1% penicillin-streptomycin for 24 h. Total RNA (500 ng) was extracted from PBMCs with TRIzol reagent (Nordic Bioscience, Beijing, China) and reverse into cDNA (10 uL) by reverse transcription kit (TakaRa, PrimeScript™ RT Master Mix, Cat No: RR036Q). The primer sequences of the specific genes (PPP1CA and CTSD) are listed in Table 1. Real-time quantitative PCR (RT-qPCR) for these mRNAs was performed and analyzed using cDNA and SYBR Green PCR Master Mix (Nordic Bioscience, Beijing, China, dilution: 5X). RT-qPCR was performed on a 7,500 fast real-time PCR system (Applied Biosystems, Foster City, CA, United States). The relative amounts of mRNA were determined based on 2^−ΔΔCT^ calculations with GAPDH as a control reference.

**TABLE 1:** Primer sequences of RT-qPCR analyses for mRNA expression.

Genes	ID	Forward	Reverse
PPP1CA	5499	ACTACGACCTTCTGCGACTAT	AGTTCTCGGGGTACTTGATCTT
CTSD	1509	ATTCAGGGCGAGTACATGATCC	CGACACCTTGAGCGTGTAG
GAPDH	2597	ACATCAAGAAGGTGGTGAAGCAG	AAAGGTGGAGGAGTGGGTGTC

### Data collection

Gene expression profiles for training cohort (GSE15932) were extracted from the Gene Expression Omnibus (GEO) database (https://www.ncbi.nlm.nih.gov/geo/) (GPL570 platform), which comprised peripheral blood samples from 8 T2DM patients and 8 controls. The GSE26168 dataset (GPL6883 platform), containing transcriptomic data from 9 T2DM patients and 8 healthy controls, was downloaded from the same database for external validation. A total of 1,519 asparagine-related genes (ARGs) were identified by merging two independent sources: 326 genes from Molecular Signatures Database (MSigDB, https://www.gsea-msigdb.org/gsea/msigdb) and 1,322 genes from GeneCards (https://www.genecards.org/) with relevance scores >6 (Li et al., 2024) (Supplementary Table S1).

### Differential expression analysis

Differentially expressed genes (DEGs) between T2DM samples and controls were identified from the training cohort (GSE15932) using the R package “limma” (v 3.54.0) (Ritchie et al., 2015) (|log_2_ fold change (FC)| >0.5, *P* < 0.05). To visualize the distribution of DEGs, a volcano plot was created with “ggplot2” package (v 3.4.4) (Gustavsson et al., 2022). The top 10 upregulated/downregulated DEGs (ranked by |log_2_FC|) were labeled on the volcano plot. Additionally, a heatmap was constructed using the R package “Complex Heatmap” (v 2.14.0) (Gu et al., 2016) to display the expression patterns of these top 20 DEGs across all samples. To obtain candidate genes associated with both asparagine and T2DM pathogenesis, the intersection of DEGs and ARGs was implemented with “Venn Diagram” package (v 1.7.3).

### Gene ontology (GO) and kyoto encyclopedia of genes and genomes (KEGG) pathway enrichment analyses

To shed light on the biological functions and signaling pathways associated with the candidate genes, GO and KEGG pathway enrichment analyses were executed with “cluster Profiler” package (v 4.7.1.003) (Wu et al., 2021). The GO analysis categorized gene functions into biological process (BP), cellular component (CC), and molecular function (MF). KEGG pathway analysis was conducted to detect metabolic and signal transduction pathways linked to the candidate genes. For both analyses, a significance threshold of adjusted *P* < 0.05 was implemented. Enrichment results were ranked by adjusted p-values in ascending order, and the top 5 most significant terms from each category were obtained for visualization.

### Protein-protein interaction (PPI) network

PPI network for the candidate genes was analyzed based on STRING database (https://string-db.org/) (confidence score >0.4). The resulting interaction data were visualized with Cytoscape (v 3.9.1). To identify candidate feature genes within the PPI network, four topological algorithms from the Cytoscape plugin “Cytohubba”—Maximum Clique Centrality (MCC), Edge Percolated Component (EPC), Closeness, and Betweenness—were applied. Genes with weights above the median threshold (i.e., exceeding the median value for MCC, EPC, Closeness, and Betweenness scores) were retained. The shared genes identified by all four algorithms were determined as candidate feature genes using the R package “ggvenn” (v 0.1.9) (Gao et al., 2021).

### Machine learning

To detect feature genes associated with asparagine in T2DM, the Boruta algorithm was implemented using the R package “Boruta” (v 8.0.0) (Zhou et al., 2023). The training cohort (GSE15932) was subjected to iterative feature importance analysis with a significance threshold of p = 0.001 and a maximum iteration parameter of maxRuns = 100. Genes classified as “Confirmed” by the Boruta algorithm—indicating statistically significant importance in distinguishing T2DM from controls—were retained for downstream analysis. A random forest classifier was constructed using “random Forest” package (v 4.7.1.1) (Alderden et al., 2018) to further refine feature selection. The model utilized 5-fold cross-validation, with the out-of-bag (OOB) error rate serving as the performance metric to determine the optimal number of decision trees (ntree). The ntree value corresponding to the lowest OOB error rate was selected to finalize the model. Genes ranked in the top 50% of mean decrease Gini importance scores were defined as feature genes. Support Vector Machine Recursive Feature Elimination (SVM-RFE) was executed employing “caret” package (v 6.0-93). The algorithm iteratively removed the least important features from the candidate gene set based on linear kernel SVM weights. A 5-fold cross-validation strategy was applied to evaluate classification accuracy, and the gene subset achieving the lowest error rate was identified. The overlapping genes across all three machine learning methods were defined as feature genes.

### Expression analysis

To validate the expression consistency of feature genes across cohorts, Wilcoxon rank-sum tests were performed separately in the training cohort (GSE15932) and validation cohort (GSE26168). Genes exhibiting statistically significant differential expression in both cohorts (*P* < 0.05), with consistent trends between T2DM and controls, were defined as biomarkers. To assess the diagnostic potential of these biomarkers, receiver operating characteristic (ROC) curves were generated. The area under the curve (AUC) was calculated to evaluate discriminatory ability between T2DM samples and controls, with AUC values interpreted as follows: AUC >0.7 indicated strong discriminatory potential. The ROC analysis was performed using the R package “pROC” (v 1.18.0).

### Gene set enrichment analysis (GSEA)

To delve into biological pathways associated with the biomarker in T2DM, GSEA was implemented with R “clusterProfiler” package (v 4.7.1.003) (|normalized enrichment score (NES)| >1, adjusted *P* < 0.05). Spearman correlation coefficients (cor) between the identified biomarkers and all other genes in the training cohort (GSE15932) were calculated with “psych” package (v 2.1.6). Genes were ordered by the cor values. The reference gene set (h.all.v2024.1.Hs.symbols.gmt) was acquired from the MSigDB.

### Immune infiltration analysis

To quantify the infiltration levels of 28 immune cell types in peripheral blood samples from training cohort (GSE15932) (Zhang Y. et al., 2023), ssGSEA was executed employing “GSVA” package (v 1.46.0) (Hänzelmann et al., 2013). A heatmap depicting immune cell infiltration scores across T2DM samples and controls was generated with “pheatmap” package (v 1.0.12). Wilcoxon tests were used to contrast immune cell enrichment scores between T2DM samples and controls. Immune cell types with adjusted *P* < 0.05 were defined as differentially infiltrated immune cells. Boxplots visualizing these differences were constructed with “ggplot2” package (v 3.4.4). Cor values between differentially infiltrated immune cells were calculated (|cor| >0.3, *P* < 0.05). A correlation heatmap was created with “ggcorrplot” package (v 0.1.4). Spearman correlation analysis was executed using “psych” package (v 2.1.6) to assess relationships between differentially infiltrated immune cells and biomarkers. Cor values and p-values were calculated (|cor| >0.3, *P* < 0.05).

### Subcellular localization prediction of biomarkers

Protein sequences corresponding to the identified biomarkers were acquired from the National Center for Biotechnology Information (NCBI) database (https://www.ncbi.nlm.nih.gov/). The subcellular localization of biomarker-associated mRNAs was predicted based on mRNALocater (http://bio-bigdata.cn/mRNALocater/), a machine learning-based database specialized in eukaryotic mRNA localization. The distribution of predicted subcellular localizations was visualized with “ggplot2” package (v 3.4.4).

### GeneMANIA network construction

Functional associations between the identified biomarkers and other genes were analyzed based on the GeneMANIA database (https://genemania.org/), which integrates heterogeneous genomic and proteomic data, including protein-protein interactions, co-expression networks, genetic interactions, and pathway associations. Biomarkers were uploaded to GeneMANIA, and the 20 genes with the highest functional similarity scores (based on weighted interaction evidence) were selected. Enriched pathways and biological functions associated with the biomarker-top 20 gene network were identified (p < 0.05, false discovery rate (FDR) <0.05). The top 7 most significant pathways and functions were retained for visualization.

### Regulation network analysis

Transcription factors (TFs) regulating the identified biomarkers were retrieved from ChIPBase database (http://rna.sysu.edu.cn/chipbase/). In addition, microRNA (miRNA) targeting the biomarkers was predicted using MicroCosm database (https://tools4mirs.org/software/mirna_databases/microcosm-targets/). Subsequently, long non-coding RNAs (lncRNAs) regulating identified miRNAs were predicted employing ENCORI database (https://rnasysu.com/encori/). Interactions with a number of supporting CLIP-seq experiments (clipExpNum) >20 were retained to ensure high-confidence lncRNA-miRNA pairs. A lncRNA-miRNA-mRNA regulatory network was created by integrating miRNA-mRNA and lncRNA-miRNA interaction pairs. The networks were visualized with Cytoscape software (v 3.9.1).

### Drug prediction and molecular docking

Potential therapeutic compounds targeting the biomarkers were detected using Comparative Toxicogenomics Database (CTD, https://ctdbase.org/). Direct and indirect drug-target interactions were retrieved (combined score >50). A network was constructed for visualization employing Cytoscape software (v 3.9.1). 3D structures of the top-ranked drugs (ranked by combined score) were downloaded in SDF format from NCBI PubChem Compound database (https://pubchem.ncbi.nlm.nih.gov/). Crystal structures of biomarker encoding proteins were obtained from UniProt database (https://www.uniprot.org/) in PDB format. Molecular docking was performed using CB-Dock2 (https://cadd.labshare.cn/cb-dock2/). The optimal binding conformation of the protein and drug was visualized using PyMOL software (v 3.1) (Seeliger and de Groot, 2010).

### Statistical analysis

Bioinformatics analyses were executed utilizing the R software (v 4.2.2). Comparative analysis of data from different groups was carried out using the Wilcoxon test (*P* < 0.05).

## Results

### Asparagine-associated genes involved in T2DM pathogenesis

**FIGURE 1:**
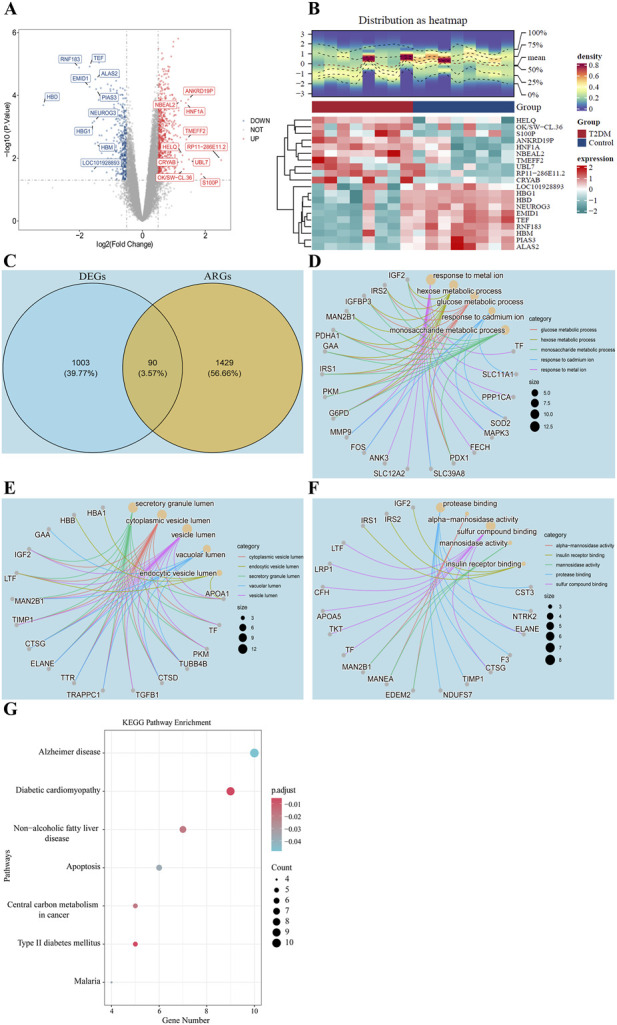
Identification of candidate genes associated with asparagine in T2DM pathogenesis. **(A)** Volcano plot of DEGs in GSE15932 (Top 10 marked); **(B)** Distribution of DEGs in T2DM and healthy control group; **(C)** Venn plot of DEGs and ARGs; **(D)** BP analysis of 90 candidate genes; **(E)** CC analysis of 90 candidate genes; **(F)** MF analysis of 90 candidate genes; **(G)** KEGG pathway enrichment of 90 candidate genes.

Transcriptome profiling revealed 1,093 DEGs between T2DM samples and controls (|log_2_FC| >0.5, *P* < 0.05), with 692 genes upregulated and 401 downregulated in T2DM samples (Figures 1A,B). Intersection analysis of these DEGs with 1,519 ARGs identified 90 candidate genes potentially involved in T2DM pathogenesis (Figure 1C). Functional annotation through GO enrichment analysis demonstrated significant associations across three categories. Candidate genes were predominantly enriched in some BP, such as glucose metabolic process, hexose metabolic process, and monosaccharide metabolic process (Figure 1D). CC involved vesicle-related structures such as cytoplasmic vesicle lumen, endocytic vesicle lumen, and secretory granule lumen (Figure 1E). For MF, candidate genes were significantly enriched in alpha-mannosidase activity, insulin receptor binding, and mannosidase activity (Figure 1F; Supplementary Table S2). KEGG pathway analysis identified 7 significantly enriched metabolic and disease pathways. Notably, the candidate genes were associated with Type II diabetes mellitus, diabetic cardiomyopathy, and Non−alcoholic fatty liver disease (Figure 1G; Supplementary Table S3). These findings strongly indicate that the regulation of asparagine could play a pivotal role in orchestrating the progression of T2DM through specific candidate genes.

### Biomarkers associated with asparagine in T2DM

**FIGURE 2:**
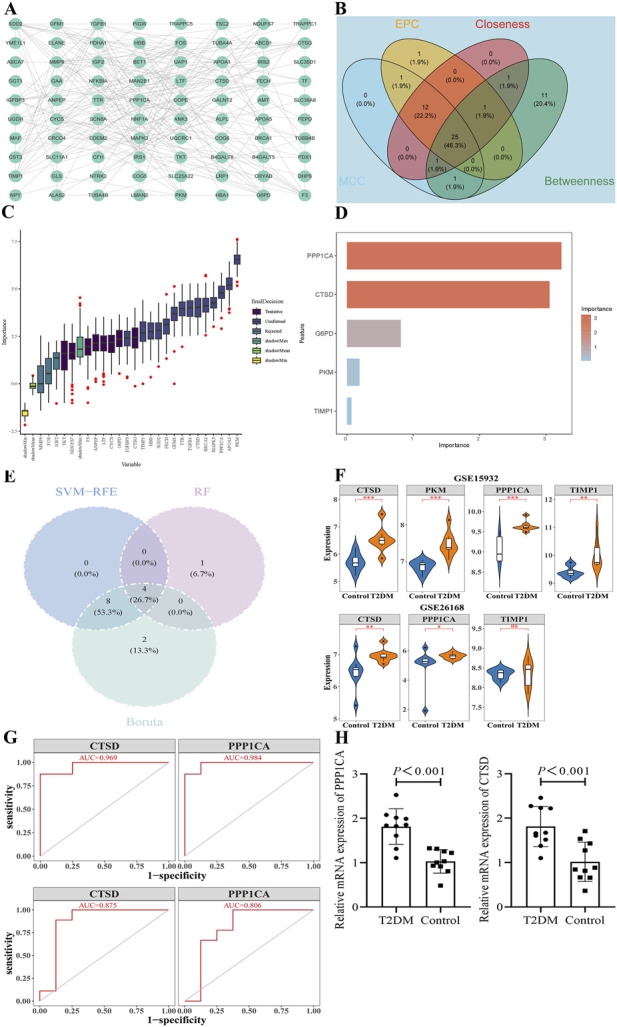
Identification of biomarkers associated with asparagine in T2DM. **(A)** PPI network analysis of 80 candidate genes; **(B)** Subsequent integration of four algorithmic approaches (EPC, Closeness, betweenness, MCC) for candidate feature genes; **(C)** Boruta algorithm plot; **(D)** Permutation feature importance with optimal ntree = 6 (minimum error rate); **(E)** 4 shared feature genes between three algorithmic outputs yielded; **(F)** Violin plot of PPP1CA, CTSD, PKM, TIMP1 expression; **(G)** ROC analysis of PPP1CA and CTSD in the training cohort (GSE15932) and the validation cohort (GSE26168); **(H)** Relative mRNA expression of PPP1CA and CTSD in the T2DM and the healthy control (n = 10).

PPI network analysis of 80 candidate genes (excluding 10 outlier proteins) revealed 813 interaction pairs, with SOD2, TGFB1, and MMP9 exhibiting the highest connectivity (Figure 2A). Subsequent integration of four algorithmic approaches identified 25 candidate feature genes (Figure 2B). Boruta algorithm further refined this set to 16 robust features, including APOA1, BRCA1, CTSD, FECH, GFM1, HBB, IGFBP3, MAPK3, PKM, PPP1CA, SOD2, TGFB1, TIMP1, and TTR (Figure 2C). Permutation feature importance with optimal ntree = 6 (minimum error rate) prioritized 5 feature genes, including CTSD, G6PD, PKM, PPP1CA, and TIMP1 (Figure 2D). The intersection of these three algorithmic outputs yielded 4 shared feature genes (PPP1CA, CTSD, PKM, TIMP1) (Figure 2E). In the training cohort (GSE15932), all four feature genes exhibited significant upregulation in T2DM samples compared to controls (*P* < 0.01). Subsequent validation in the independent cohort (GSE26168) revealed that PPP1CA and CTSD maintained consistent overexpression patterns in T2DM samples (*P* < 0.05). In contrast, TIMP1 showed nonsignificant expression trends, while PKM was excluded from analysis due to its absence in the validation cohort (Figure 2F). To further assess the discriminatory potential of PPP1CA and CTSD, ROC analysis was performed. In the training cohort (GSE15932), PPP1CA achieved an AUC of 0.969 and CTSD an AUC of 0.984, both exceeding the threshold of 0.9 indicative of strong diagnostic utility. In the validation cohort (GSE26168), PPP1CA achieved an AUC of 0.806 and CTSD an AUC of 0.875, where both genes again demonstrated AUC values >0.8 (Figure 2G). Then, mRNA expression of PPP1CA and CTSD was validated by RT-qPCR. Results indicated PPP1CA and CTSD were significantly upregulated in T2DM samples (*P* < 0.001, Figure 2H). These results establish PPP1CA and CTSD as robust biomarkers for T2DM.

### Functional pathway enrichment revealed distinct roles of PPP1CA and CTSD in T2DM pathogenesis

**FIGURE 3:**
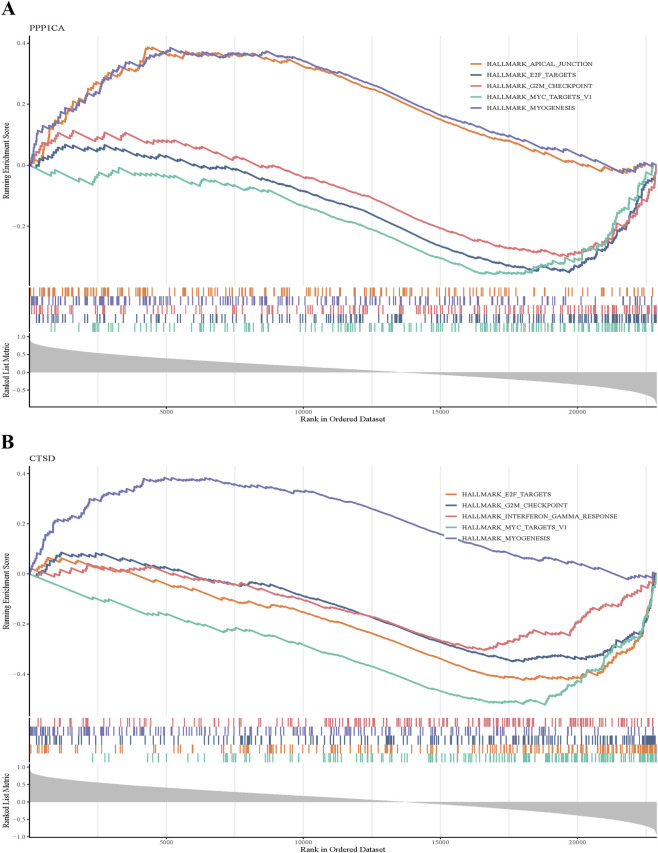
GSEA of PPP1CA and CTSD in T2DM. **(A)** GSEA of PPP1CA in T2DM; **(B)** GSEA of CTSD in T2DM.

To elucidate the biological pathways associated with PPP1CA and CTSD, GSEA was systematically performed. PPP1CA demonstrated significant enrichment in 8 hallmark pathways (|NES| >1, adjusted *P* < 0.05), with prominent involvement in MYC targets V1, E2F targets, G2M checkpoint, and myogenesis (Figure 3A; Supplementary Table S4). For CTSD, significant pathway enrichment was observed in myogenesis, G2M checkpoint, E2F targets, MYC targets V1 (Figure 3B; Supplementary Table S5). These results collectively highlight distinct yet complementary functional roles of PPP1CA and CTSD in T2DM progression.

### Immune microenvironment remodeling in T2DM progression

**FIGURE 4:**
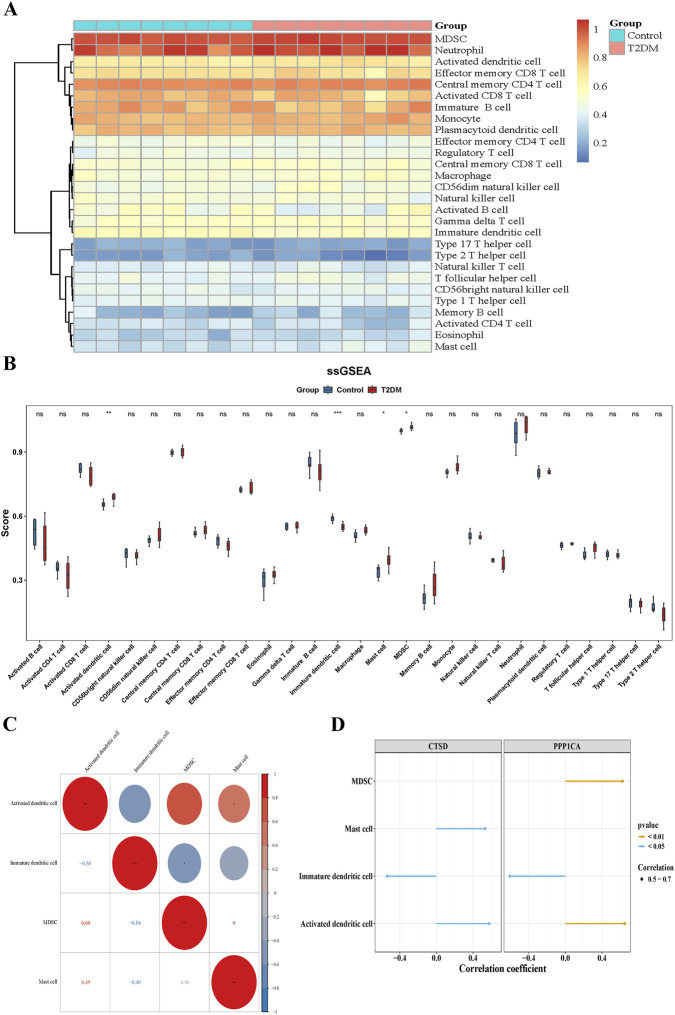
Immune microenvironment analysis of T2DM progression. **(A)** Hotmap of immune-infiltrating cells between T2DM and healthy control group; **(B)** ssGSEA of immune-infiltrating cells scores between T2DM and healthy control group; **(C)** Correlation analysis of immune-infiltrating cells; **(D)** Correlation analysis of immune-infiltrating cells with PPP1CA and CTSD.

Immune infiltration analysis unveiled significant alterations in immune cell composition between T2DM samples and controls. Activated dendritic cells (DCs, *P* < 0.01), mast cells (*P* < 0.05), and myeloid-derived suppressor cells (MDSCs) (*P* < 0.05) exhibited elevated infiltration levels in T2DM samples compared to controls, while immature DCs showed marked depletion (*P* < 0.001, Figures 4A,B). Correlation analysis demonstrated a strong positive association between mast cells and activated DCs (cor = 0.49, *P* < 0.05), whereas MDSCs inversely correlated with immature DCs (cor = −0.54, *P* < 0.05) (Figure 4C). Notably, PPP1CA and CTSD displayed distinct immunomodulatory associations. PPP1CA was negatively correlated with immature DCs infiltration (cor = −0.61, *P* < 0.05) but positively correlated with activated DCs (cor = 0.65, *P* < 0.01) and MDSCs (cor = 0.62, *P* < 0.01). CTSD showed positive correlations with activated DCs (cor = 0.58, *P* < 0.05) and mast cells (cor = 0.53, *P* < 0.05), alongside a negative correlation with immature DCs (cor = −0.54, *P* < 0.05) (Figure 4D; Supplementary Table S6). These findings collectively indicated systemic remodeling of the immune microenvironment in T2DM. The biomarker-immunocyte correlations suggested that PPP1CA and CTSD might orchestrate immune-metabolic crosstalk, potentially linking asparagine metabolism to T2DM progression.

### Subcellular localization and multi-layer regulatory networks revealed functional roles of PPP1CA and CTSD in T2DM

**FIGURE 5:**
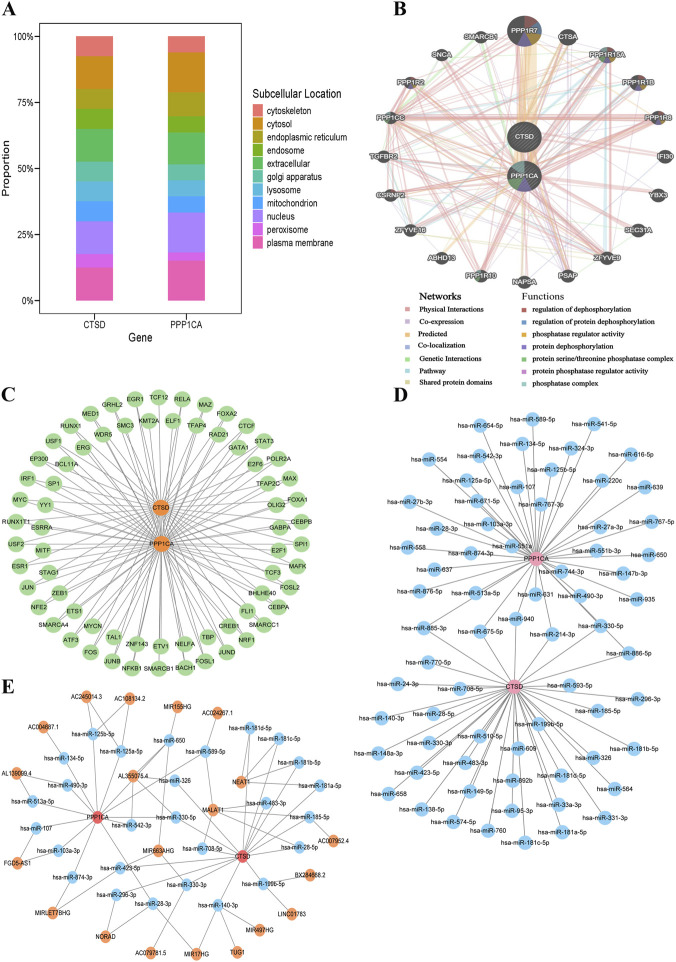
Subcellular localization and multi-layer regulatory network analysis of PPP1CA and CTSD in T2DM. **(A)** Subcellular localization of PPP1CA and CTSD; **(B)** GeneMANIA network analysis of PPP1CA and CTSD; **(C)** Transcriptional regulation analysis of PPP1CA and CTSD; **(D)** miRNA-mRNA network analysis of PPP1CA and CTSD; **(E)** lncRNAs networks of PPP1CA and CTSD.

Subcellular localization analysis revealed distinct compartmentalization patterns for the biomarkers. CTSD exhibited balanced distribution across cytoplasmic matrix, extracellular space, nucleus, and plasma membrane, with moderate presence in lysosomes, endoplasmic reticulum, Golgi apparatus, endosomes, cytoskeleton, and mitochondria, and minimal peroxisomal localization. In contrast, PPP1CA demonstrated predominant localization in cytoplasmic matrix, nucleus, and plasma membrane, followed by extracellular space and endoplasmic reticulum (Figure 5A). GeneMANIA network analysis revealed PPP1CA and CTSD primarily interacted with co-expressed genes through physical interactions, co-expression, predicted, and co-localization. Functional enrichment of these interaction partners demonstrated significant involvement in phosphatase-related processes, such as regulation of dephosphorylation, regulation of protein dephosphorylation, phosphatase regulator activity, protein dephosphorylation, protein serine/threonine phosphatase complex formation, protein phosphatase regulator activity, and phosphatase complex assembly (Figure 5B). PPP1CA demonstrated binding capacity with 69 TFs, while CTSD interacted with 38 TFs. Notably, RUNX1T1, MYC, and IRF1 exhibited dual regulatory control over both PPP1CA and CTSD, suggesting potential shared transcriptional mechanisms in T2DM pathophysiology (Figure 5C). MicroRNA interaction profiling identified 75 miRNA-mRNA regulatory pairs, with PPP1CA targeted by 39 miRNAs and CTSD by 36 miRNAs. The conserved miRNAs hsa-miR-885-3p, hsa-miR-675-5p, and hsa-miR-940 emerged as co-regulators of both biomarkers, indicating potential post-transcriptional coordination (Figure 5D). Exploration of competing endogenous RNA networks revealed lncRNA-mediated regulatory axes. The lncRNA AL355075.4 was predicted to simultaneously regulate both PPP1CA and CTSD through competitive binding to hsa-miR-330-5p, forming the AL355075.4-hsa-miR-330-5p-PPP1CA and AL355075.4-hsa-miR-330-5p-CTSD regulatory axes (Figure 5E). Collectively, these results delineated multi-layered regulatory networks governing PPP1CA and CTSD expression, encompassing transcriptional, post-transcriptional, and competing endogenous RNA-mediated mechanisms, which might critically influence T2DM pathogenesis.

### Norcantharidin and naringenin are pharmacological modulators for PPP1CA and CTSD

**FIGURE 6:**
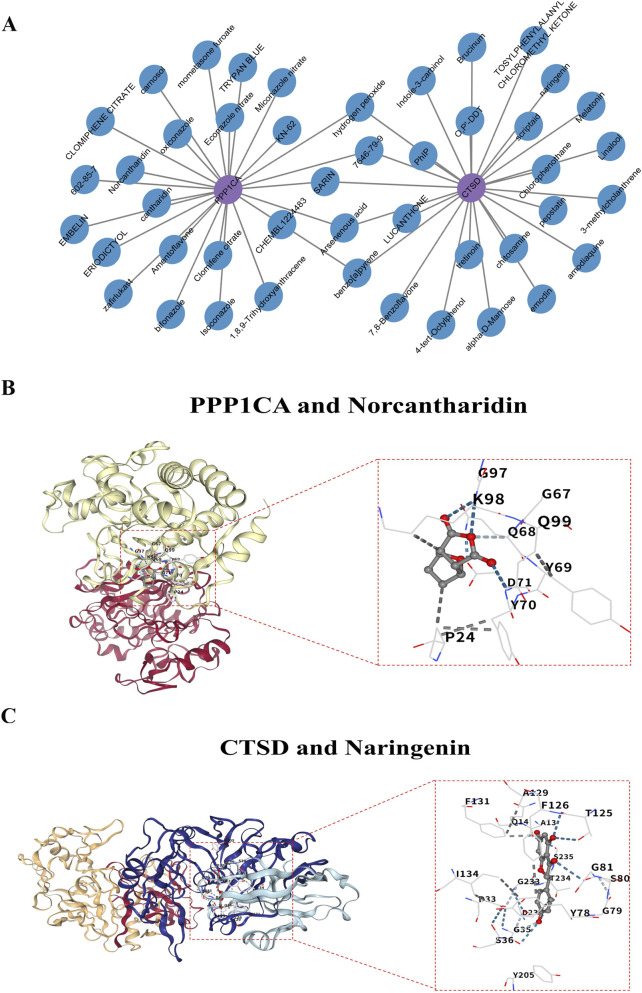
Pharmacological compounds analysis of PPP1CA and CTSD. **(A)** Pharmacological compounds network analysis for PPP1CA and CTSD; **(B)** Molecular docking of norcantharidin and PPP1CA; **(C)** Molecular docking of naringenin and CTSD.

To explore therapeutic candidates targeting the identified biomarkers, pharmacological profiling was performed. A total of 103 compounds were predicted to interact with PPP1CA and CTSD, with 72 compounds specifically targeting PPP1CA and 31 targeting CTSD. Among these, norcantharidin was identified as a specific ligand for PPP1CA, while naringenin exhibited exclusive targeting toward CTSD. Notably, sarin, benzo[a]pyrene, and hydrogen peroxide demonstrated dual targeting capabilities for both biomarkers (Figure 6A). Molecular docking simulations were conducted to validate binding interactions between the biomarkers and their top-scoring ligands. PPP1CA showed the strongest affinity for norcantharidin, with a binding energy of −6.2 kcal/mol (Table 2; Figure 6B), with key interactions with pocket 2 (primarily involves residues on protein chain B, such as PRO24, TYR69, and ASP71). Similarly, CTSD exhibited optimal structural compatibility with naringenin, achieving a binding energy of −7.4 kcal/mol (Table 2; Figure 6C), with key interactions with pocket 1 (primarily involves residues on protein chains C and D, including TYR78, HIS77, ASP33, ILE124, PHE126). The favorable binding energies observed in molecular docking supported the therapeutic potential of these compounds as modulators of PPP1CA and CTSD activity in T2DM pathogenesis.

**TABLE 2:** Binding energy of norcantharidin, naringenin to PPP1CA and CTSD.

Target	Compounds	Binding affinity (kcal/mol)
PPP1CA	Norcantharidin	−6.2
CTSD	Naringenin	−7.4

## Discussion

The underlying molecular mechanisms of T2DM remained elusive. Meanwhile, the functional roles of asparagine and its related biomarkers in T2DM pathogenesis were still unclear and had not been explored before. In this study, we successfully identified PPP1CA and CTSD as core biomarkers associated with asparagine in the pathogenesis of T2DM through the integration of transcriptome data and machine learning, and validated their significant upregulation in T2DM with high diagnostic accuracy. Furthermore, our study revealed the critical roles of PPP1CA and CTSD in glucose metabolism, insulin receptor binding, and immune function, with notable enrichment in E2F targets, G2M checkpoint, and myogenesis pathway. Additionally, we found substantial alterations in the immune cell composition of T2DM patients, including increased infiltration of activated DCs, mast cells, and MDSCs. We revealed that PPP1CA was negatively correlated with immature DCs infiltration but positively correlated with activated DCs and MDSCs. CTSD showed positive correlations with activated DCs and mast cells, alongside a negative correlation with immature DCs. Moreover, we constructed a multi-level regulatory network involving TFs, miRNAs, and lncRNAs, which elucidates the complex regulatory mechanisms governing PPP1CA and CTSD. Finally, we identified several compounds—such as norcantharidin and naringenin—with high binding affinity to PPP1CA and CTSD, providing valuable evidence for clinical treatment and drug development in T2DM.

We successfully identified PPP1CA and CTSD as core biomarkers associated with asparagine in T2DM pathogenesis and validated their critical roles in glucose metabolism, insulin receptor binding, and immune function. PPP1CA is one of the core catalytic subunits of protein phosphatase 1 (PP1), involved in regulating glycogen metabolism, muscle contractility, and protein synthesis (Hoermann et al., 2020). The role of PPP1CA in T2DM is primarily linked to the dephosphorylation of AKT (also known as protein kinase B, PKB) and AMPK, which leads to a suppression of their activity (Huang et al., 2024). The AKT/PKB signaling pathway has a very important role in insulin resistance, glucose intolerance and glucose transportation. The pivotal role of AKT signaling in systemic glucose homeostasis is unequivocally demonstrated. Previous study demonstrated that targeted deletion of AKT isoforms results in insulin resistance and glucose intolerance (Cho et al., 2001). Mutations of AKT have been identified in patients with severe insulin resistance (George et al., 2004). PP1 is a major phosphatase that dephosphorylates AKT. PP1 and AKT can form an interacting complex, negatively regulating AKT activation (Xiao et al., 2010). Therefore, the downregulation of AKT results in reduced glycogen synthesis in the liver, insulin resistance and glucose intolerance in T2DM. Furthermore, PPP1CA also interacts with CARM1 to limit the effects of AMPK on glucose metabolism, and thus participates in the regulation of metabolism and metabolic reprogramming (Zhang L. et al., 2023). CTSD is a member of the cathepsin superfamily lysosome, residing aspartic protease that plays an important role in maintaining tissue homeostasis and metabolism (Benes et al., 2008). It has also been reported to be involved in the pathogenesis of T2DM (Ding et al., 2020a). Previous study has indicated higher CTSD levels in patients with T2DM and severity of diabetic retinopathy (Reddy et al., 2017). CTSD has been implicated in the regulation of insulin-like growth factors (IGFs) bioavailability, a process strongly associated with the development of insulin resistance. Functioning as a key metabolic node, CTSD both responds to various metabolic signals and modulates glucose metabolism through insulin and IGF-dependent pathways (Ding et al., 2020b). A key finding from prior research is that the suppression of circulating CTSD activity results in decreased plasma insulin levels in rats (Khurana et al., 2019). Those results support that PPP1CA and CTSD are potential therapeutic targets for T2DM. Furthermore, the identified biomarkers associated with asparagine offer valuable support and breakthrough directions for early diagnosis, disease progression monitoring, and targeted therapeutic development for T2DM.

GSEA analysis revealed that the expression of both PPP1CA and CTSD is significantly associated with the E2F targets pathway. The E2F transcription factor family serves as a core regulator controlling the transition from the G1 to S phase of the cell cycle (Ohtani, 1999; Bogdanow et al., 2021). In mature pancreatic β-cells, E2F activity is tightly regulated to maintain insulin secretion (Bourouh et al., 2022). However, aberrant or sustained activation of the E2F pathway can force β-cells to exit quiescence and re-enter the replication cycle, leading to their functional de-differentiation. This is characterized by downregulation of insulin gene expression, loss of glucose sensitivity, and impaired expression of transcription factors (Oger et al., 2023). Obviously, excessive E2F activation not only fails to promote β-cell proliferation but also impairs their secretory function. PPP1CA may indirectly relieve Rb-mediated suppression of E2F by dephosphorylating and activating inhibitors of Rb, thereby promoting transcriptional activation of E2F target genes (Berndt et al., 1997; Liu et al., 2006). Conversely, deficiency of CTSD has been shown to impair cell cycle progression (Secomandi et al., 2022). Therefore, elevated CTSD expression may reflect its role in providing necessary proteolytic and signaling support to meet the high metabolic and replicative demands driven by E2F activation. Therefore, PPP1CA and CTSD may cooperatively disrupt cell cycle homeostasis in pancreatic β-cells through PPP1CA-mediated dephosphorylation of Rb-E2F pathway and CTSD-maintained essential metabolic environment mechanisms, inducing functional inactivation and dedifferentiation, and ultimately leading to defective insulin secretion.

Our results revealed substantial alterations in the immune cell landscape of patients with T2DM, characterized by elevated infiltration of activated DCs, mast cells, and MDSCs. We revealed that PPP1CA was negatively correlated with immature DCs infiltration but positively correlated with activated DCs and MDSCs. CTSD showed positive correlations with activated DCs and mast cells, alongside a negative correlation with immature DCs. DCs function as professional antigen-presenting cells with the dual capacity to instigate and suppress immune reactions, thereby serving as a crucial bridge between the innate and adaptive arms of the immune system. The maturation of immature DCs, which is induced by damage- or pathogen-associated molecular patterns, is defined by a marked upregulation of surface markers and the release of cytokines that are indispensable for the priming and activation of naïve T lymphocytes, thereby catalyzing antigen-specific adaptive immune responses (Sundara Rajan and Longhi, 2016). In addition to this immunostimulatory role, DCs are instrumental in the establishment and maintenance of immunological tolerance. Given their ability to modulate the functions of both T cells and macrophages, DCs are considered pivotal in orchestrating the recruitment of other immune cells to sites of immune response. Chronic low-grade inflammation characterized by increased accumulation of immune cells, especially macrophages and T lymphocytes, in adipose tissue is one of the main mechanisms associated with IR and T2DM (Esser et al., 2014). A potential explanation for the increased accumulation of activated DCs in patients with T2DM may be driven by elevated pro-inflammatory cytokines (such as tumor necrosis factor-α [TNF-α], interleukin 6 [IL-6], interleukin 1β [IL-1β]) and adipose tissue inflammation (Soedono and Cho, 2021). Meanwhile, we revealed that the asparagine-associated biomarkers, PPP1CA and CTSD are positively correlated with activated DCs. This correlation is mechanistically linked to the pro-inflammatory cytokine production regulated by PPP1CA and CTSD (Clavarino et al., 2012; Zou et al., 2013). In addition, we found that MDSCs also increase in T2DM patients. MDSCs consist of a diverse group of immature myeloid cells that originate from the bone marrow and serve as essential immunosuppressive agents by suppressing T cell and other immune cell functions. The accumulation of MDSCs enhances insulin response; conversely, the depletion of MDSCs results in reduced glucose tolerance and IR (Wang et al., 2021). The accumulation of MDSCs in patients with T2DM is mechanistically linked to a milieu of chronic inflammation and its associated chemokines and cytokines. Chemokines and cytokines present in chronic inflammation can recruit MDSCs from peripheral circulation and bone marrow to the inflammation site, adipose tissue and visceral fat (Ghemiş et al., 2024). Specifically, the recruitment of monocytic MDSC subsets are regulated by CC-chemokine ligand 2 (CCL2), whereas polymorphonuclear MDSCs are recruited by CCL5. This result is supported by clinical evidence, as the concentrations of both CCL2 and CCL5 have been reported to be significantly elevated in T2DM patients compared to healthy controls (Pan et al., 2021; Wu and Ma, 2024). These findings propose an integrated analysis wherein dysregulated metabolic pathways and chronic inflammation orchestrate a unique immune cell profile, featuring concurrent immune activation and suppression, which contributes to the immunometabolic dysfunction in T2DM.

Subcellular localization analysis revealed CTSD exhibited balanced distribution across cytoplasmic matrix, extracellular space, nucleus, and plasma membrane, aligns with its known multifunctional roles in proteolysis, autophagy, and signal transduction (Di et al., 2021; Jiang et al., 2025). This suggests that CTSD may influence metabolic and inflammatory pathways in T2DM at multiple aspects, potentially through degrading specific substrates or activating precursor molecules in distinct cellular compartments. In contrast, PPP1CA demonstrated predominant localization in cytoplasmic matrix, nucleus, and plasma membrane, consistent with its core function as a catalytic subunit of serine/threonine protein phosphatases. It regulates the activity of metabolism-related kinases such as AKT and AMPK in the cytoplasm, while potentially directly influencing gene expression programs in the nucleus by dephosphorylating transcription factors or histones (Xiao et al., 2010). GeneMANIA network analysis identified three TFs: RUNX1T1, MYC, and IRF1, capable of co-regulating PPP1CA and CTSD. Among them, MYC, a master regulator of metabolism and proliferation (Adiamah et al., 2025; Staal et al., 2016), may form a pathway linking hypermetabolism to immune-inflammatory activation by simultaneously activating the expression of PPP1CA and CTSD. At the post-transcriptional level, miRNAs such as hsa-miR-885-3p, hsa-miR-675-5p, and hsa-miR-940 act as shared negative regulators. Dysregulation of these miRNAs may lead to uncontrolled upregulation of PPP1CA and CTSD in T2DM.

Conclusively, this study identified PPP1CA and CTSD as asparagine-related biomarkers driving immune-metabolic crosstalk in T2DM, enhancing the understanding of the pathogenesis of asparagine metabolism in T2DM, which provided novel insights into T2DM mechanisms and potential intervention strategies. However, several limitations of this study should be noted. Firstly, the bioinformatics analysis was primarily based on public databases. Although preliminary experimental validation was conducted, the specific molecular mechanisms by which PPP1CA and CTSD influence immune cell infiltration and metabolic homeostasis need to be further elucidated through gene knockout/overexpression experiments in both cellular and animal models. Secondly, the multi-layer regulatory networks, including TFs, miRNA, lncRNA, and the efficacy of candidate drugs require further experimental validation. Future research could focus on validating the diagnostic value of PPP1CA and CTSD in larger clinical cohorts, dissecting their downstream signaling pathways in specific cell types such as β-cells and immune cells, and conducting preclinical studies to evaluate the efficacy and safety of targeted drug interventions, thereby facilitating their translation into clinical applications.

## Data Availability

The datasets presented in this study can be found in online repositories. The names of the repository/repositories and accession number(s) can be found in the article/Supplementary Material.
