# Supplementary figures and images for: Asparagine-related biomarkers and regulatory mechanisms in type 2 diabetes mellitus
Source: Front Mol Biosci. 2025 Dec 17;12:1733878. doi: 10.3389/fmolb.2025.1733878 (PMC12753446; doi:10.3389/fmolb.2025.1733878)

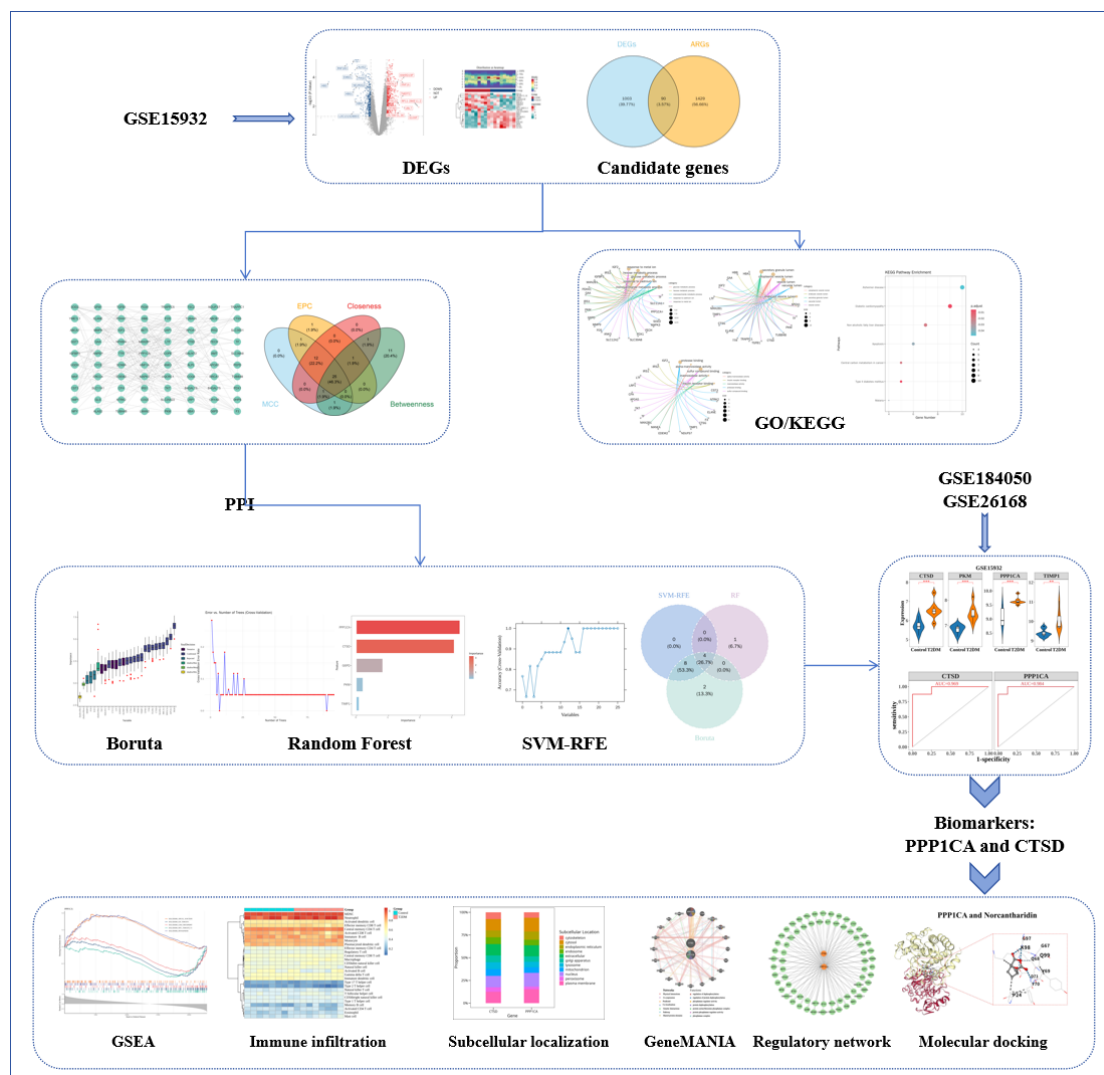

Figure S1 Analysis of the workflow

Supplement: Supplementary file 8 [file Image1.pdf]
